# Systemic Regulatory T Cells and IL-6 as Prognostic Factors for Anatomical Improvement of Uveitic Macular Edema

**DOI:** 10.3389/fimmu.2020.579005

**Published:** 2020-09-25

**Authors:** Jessica Matas, Victor Llorenç, Alex Fonollosa, David Díaz-Valle, Cristina Esquinas, Maria Teresa Sainz de la Maza, Marc Figueras-Roca, Joseba Artaraz, Barbara Berasategui, Marina Mesquida, Alfredo Adán, Blanca Molins

**Affiliations:** ^1^Group of Ocular Inflammation, Institut d’Investigacions Biomèdiques Agustí Pi i Sunyer (IDIBAPS), Hospital Clínic de Barcelona, Barcelona, Spain; ^2^Department of Ophthalmology, BioCruces Health Research Institute, Hospital Cruces, University of the Basque Country, Baracaldo, Spain; ^3^Ophthalmology Department and Health Research Institute (IdISSC), Hospital Clinic of San Carlos, Madrid, Spain; ^4^Valle Hebron Research Institute, Autonomous University of Barcelona, Barcelona, Spain

**Keywords:** macular edema, uveitis, cytokines, regulatory T cells, biomarkers

## Abstract

**Purpose:**

To investigate whether systemic immune mediators and circulating regulatory T cells (Tregs) could be prognostic factors for anatomic outcomes in macular edema secondary to non-infectious uveitis (UME).

**Methods:**

Multicenter, prospective, observational, 12-month follow-up study of 60 patients with UME. Macular edema was defined as central subfield thickness (CST) > 300 μm measured with spectral domain optical coherence tomography (SD-OCT). Serum samples and peripheral blood mononuclear cells (PBMC) were obtained from venous blood extraction at baseline. Serum levels of IL-1β, IL-6, IL-8, IL-17, MCP-1, TNF-α, IL-10, and VEGF were determined by Luminex. Tregs population, defined as CD3^+^CD4^+^FoxP3^+^ in PBMC, was determined by flow cytometry. Main outcome measure was the predictive association between searched mediators and CST sustained improvement, defined as CST < 300 microns or a 20% CST decrease, at 6 months maintained until 12-months compared to baseline levels.

**Results:**

Multivariate logistic regression analysis showed an association between CST sustained improvement at 12 months follow-up and IL-6 and Tregs baseline levels. Higher IL-6 levels were associated with less events of UME improvement (OR: 0.67, 95% CI (0.45–1.00), P = 0.042), whereas higher levels of Tregs favored such improvement (OR: 1.25, 95% CI: 1.12–2.56, P = 0.049).

**Conclusions:**

Increased levels of Tregs and reduced levels of IL-6 in serum may be prognostic factors of sustained anatomical improvement in UME. These findings could enforce the opportunity to develop more efficient and personalized therapeutic approaches to improve long-term visual prognosis in patients with UME.

## Introduction

Non-infectious uveitis accounts for 10% of cases of total blindness in developed world and may result in persistent visual impairment in untreated patients (1). Uveitis may affect people of all ages but most frequently occurs in the working age population, thus implying a high socioeconomical burden (2). Macular edema remains the most commonly found structural complication of uveitis that results in central visual impairment although only one third of uveitis patients develop it (3). The pathogenesis of uvetitic macular edema (UME) involves breakdown of the blood-retinal barrier, followed by both intracellular and extracellular fluid accumulation within the macular retina (4). However, the underlying pathophysiology as well as the molecular mediators involved are not fully understood, therefore making UME a controversial topic.

Chronic retinal inflammation has been observed in early phases and also in the sight-threatening advanced forms of UME. The uveitic retina is characterized by a complex milieu of dysregulated proinflammatory factors. Increased levels of certain proinflammatory cytokines, such as interleukin-6 (IL-6), IL-8, and tumor necrosis factor-α (TNF-α), both in serum and aqueous humor, have been found in patients with UME (5). Vascular endothelial growth factor (VEGF) may also play a major role as it can be increased in aqueous humor of patients with UME and in other forms of macular edema (5, 6). On the other hand, in the last years, the interest in regulatory T cells (Tregs) as markers of activity and modulators of autoreactive T lymphocytes in uveitis and other inflammatory diseases has gained attention (7). Patients with active uveitis have reduced circulating levels of Tregs compared to healthy subjects, with Tregs levels being significantly upregulated during quiescent disease (8, 9). Moreover, adoptive transfer of Tregs has resulted in greater protection against intraocular inflammation in experimental models of uveitis (10, 11). However, it is unknown whether Tregs are also involved in the pathophysiology of UME.

UME may persist despite adequate control of activity, thus leading to permanent photoreceptor damage and loss of central visual acuity (12). After the acute phase, UME may either subside spontaneously or respond to treatment intended to reduce local inflammatory mediators. Moreover, in relapsing and chronic forms of UME, especially in bilateral forms, systemic treatment should be started, usually consisting of corticosteroids and/or immunomodulators (13). However, despite these options, a number of patients still remain refractory to treatment and UME may lead to severe visual impairment.

In summary, there is scarce data regarding the factors influencing sustained anatomic and visual recovery in eyes with UME. A better understanding of the prognostic biomarkers in patients with UME would allow the use of more efficient and selective pharmacological approaches to improve long-term visual prognosis and minimizing unnecessary drug-induced toxicity. In this prospective study, we aimed to investigate the association of several systemic immune mediators and Tregs with the anatomic outcome of UME after 12 months of follow-up.

## Materials and Methods

### Study Design and Selection Criteria

We conducted a multicenter, prospective, observational, 12-month follow-up study to analyze the association of peripheral blood immune mediators and Tregs with the clinical evolution of UME. Adult (>18 years-old) patients with non-infectious UME in at least one eye were proposed for inclusion. UME was defined as central subfield thickness (CST) of >300 μm as measured by SD-OCT (HD-Cirrus, Carl Zeiss Meditec, Dublin, CA) and presence of fluid (intraretinal and/or subretinal) in the macula. Whenever bilateral UME was present, the eye with the higher CST value in the fovea according to OCT measurements in μm was selected as the study eye. Exclusion criteria were limited to infectious uveitis, highly hazy ocular media, concurrent ocular diseases, exclusive tractional UME, pregnancy, immunocompromising systemic diseases (including, but not limited to HIV, leukemia, lymphoma, and chemotherapy), and eyes with any intervention (intraocular surgery, laser, and intravitreal therapy) in the previous 4 months.

Three referral centers for ocular inflammatory diseases in Spain (Clínic Hospital of Barcelona, Cruces Hospital of Bilbao and Clínico San Carlos Hospital of Madrid) participated in the recruitment of patients from January 2015 to January 2017. Local ethics committees approved the study (Comité Ético de Investigación Clínica del Hospital Clínic de Barcelona 2013/8574; Comité de Ética de la Investigación con Medicamentos de Euskadi, Hospital Universitario Cruces PI201406; Comité Ético de Investigación Clínica del Hospital Clínico San Carlos de Madrid 13/244-E). All patients provided written informed consent, and the research followed the regulations of the Declaration of Helsinki.

### Data Collection and Ophthalmic Assessment

Patients were evaluated at each clinical site, and protocol-based assessments were performed at different time-points: baseline, month 1, month 6, and month 12 of follow-up. Other visits at different time-points were allowed at the discretion of the treating physician. Data were recorded in an electronic case report form designed *ad hoc*.

Medical record data from each patient included demographics (age, race, and sex), diagnosis classified by anatomic location according to the Standardization of Uveitis Nomenclature (SUN) criteria (14), laterality of disease, systemic or isolated ocular inflammatory disease classification, and a complete ophthalmic examination.

Data gathered from evaluation of each patient included best-corrected visual acuity (BCVA, with Snellen charts in decimal scale at a test distance of 6 m), the presence or absence of disease activity, categorical and quantitative OCT findings, and UME treatment. The SUN recommendations were used for anterior chamber and vitreous inflammatory activity gradations. SD-OCT exploration was used as determination of UME using Macular cube 512 × 128 A-scan, within a 6 × 6 mm area centered on the fovea. Imaging assessment was performed by two investigators who were masked to clinical data of the corresponding patients. In the event of discrepancies, the two graders made the assessment together and reached a consensus. Masked investigators were asked to determine the CST and the pattern of UME (sub-retinal fluid, cystoid, diffuse, or tractional components). Macular volume, subfoveal choroidal thickness (enhanced deep imaging mode), maximum diameter of the greatest cyst if present, subretinal fluid pocket’s length in a transfoveal B-scan were also recorded. Ancillary tests to rule out infectious origin (including but not limited to luetic serology, quantiFERON-TB Gold and toxoplasma serology) and to appropriately classify the uveitis were ordered at the investigator discretion.

### Sample Collection

Two peripheral blood samples were obtained at the time of patient inclusion in the presence of UME according to the aforementioned definition. Serum was obtained from sample 1 as follows: blood was centrifugated at 1600 g within 20 min and stored at -80°C until inflammatory mediators were determined. The levels of Tregs in peripheral blood were determined from sample 2: peripheral blood mononuclear cells (PBMCs) were obtained by Ficoll (Ficoll-Plaque Plus, GE Healthcare) gradient centrifugation, washed twice with RPMI 1640 containing 2% heat-inactivated fetal calf serum and cryopreserved for further staining and analysis by flow cytometry. Cryopreserved serum and PBMC samples were then shipped to the Ocular Inflammation Laboratory (Hospital Clinic of Barcelona) where they were analyzed.

### Cytokine Determination

Eight immune mediators were determined: IL-1β, IL-6, IL-8, IL-17, MCP-1, TNF-α, IL-10, and VEGF. These molecules were chosen based on published results of previous studies regarding both local and systemic biomarkers in uveitis and UME. Selected immune mediators were determined by a Luminex platform (Millipore’s MilliPlex Human Cytokine/Chemokine kit) used to measure cytokine and chemokine levels in serum samples using an assay plate layout consisting of seven standards in duplicate (3.2–2,000 pg/mL), one blank well (for background fluorescence subtraction), two internal quality control samples in duplicate, and 25-μL duplicates of each serum sample. The MilliPlex method was performed as recommended by the manufacturer. Zero values were statistically handled as a third of the provided detection limit.

### Treg Determination by Flow Cytometry

FoxP3 levels in CD3^+^CD4^+^ cells from cryopreserved PBMCs were determined by flow cytometry. A live/dead Fixable Dead Cell Stain (Invitrogen) was used to exclude dead cells from the analysis, followed by incubation for 30 min on ice with AF700-CD3 (clone OKT3) and FITC-CD4 (OKT4) (eBiosciences). Cells were then washed, fixed and permeabilized before adding PE-FOXP3 (clone 236A/E7) (eBiosciences) for 30 min. Appropriate isotype controls were included in each experiment. Flow cytometric analyses were performed on a Fortessa flow cytometer (BD Biosciences) with a total of 50,000 events recorded for each sample through a live CD3^+^CD4^+^ lymphocyte gate. Analysis of flow cytometry data was performed using FlowJo software. Tregs were defined as CD3^+^CD4^+^FoxP3^+^ cells.

### Outcome Measures

To determine whether Tregs and serum immune mediators can be predictive factors for anatomic outcomes in UME, we analyzed the association between baseline levels of the above-mentioned immune mediators and Tregs with sustained anatomical improvement of UME. Improvement of UME was defined as a 20% CST decrease or CST < 300 µm at 6 months and maintained at 12 months of follow-up compared to baseline.

### Statistical Analysis

To describe the qualitative variables, absolute frequencies and percentages were used. The description of quantitative variables was performed using the mean and standard deviation (SD). The Kolmogorov-Smirnov test was used to assess the normality of distributions. In the case of quantitative variables, the comparison of the characteristics of the eyes depending on the presence of sustained anatomical improvement was carried out using the Student t-test (or Mann-Whitney U-test if normality was not assumed). The Chi-squared test (Fisher test for frequencies < 5) was used for the comparison of categorical variables. Wilcoxon test was performed in order to analyze changes in CST during follow up.

A back stepwise logistic regression analysis was developed including the defined and CST-based favorable anatomical outcome item as the dependent variable, and as independent variables, those cytokines and clinical variables with a P-value < 0.2 in the univariate analysis and clinical pertinence (evolution time of the EMU to baseline, age at baseline, and sex). The results have been described with odds ratios (OR), 95% confidence intervals (CI), and P-values. Linear relationships between defined biomarkers were assessed using Spearman tests. For all tests, values of P <0.05 were considered statistically significant. The R Studio statistical package (version 2.5) was used for statistical analysis.

## Results

Sixty eyes of sixty patients were included. The mean age of the group was 51.1 years (SD, 15.1; range, 21–89). The mean previous duration of UME at the inclusion time was 17.37 months (SD, 31.5; range, 0–144 months). **Table 1** shows patients’ demographics and clinical data. The description of etiological diagnosis showed that systemic disorders were the predominant ones (n = 24), followed by isolated ocular syndromes (n = 21), and unclassified uveitis (n = 15).

**Table 1:** Baseline ophthalmic characteristics in eyes with uveitic macular edema.

Baseline characteristics	
Eyes/Patients (n/n)	60/60
Evolution time to baseline (months, mean ± SD)	17 ± 31
Follow-up (months)	12
Age (years, mean ± SD)	51 ± 15
Female, n (%)	35 (58.3)
Bilateral, n (%)	23 (38.3)
Patterns of UME^1^	
Cystoid, n (%)	47 (78.3)
Diffuse, n (%)	14 (23.3)
Subretinal fluid, n (%)	21 (35)
Tractional component^2^, n (%)	18 (30)
Anatomical classification (SUN)	
Anterior, n (%)	16 (26.6)
Intermediate, n (%)	9 (15)
Posterior, n (%)	22 (36.7)
Panuveitis, n (%)	13 (21.7)
Etiologic classification	
Unclassifiable (undifferentiated), n (%)	15 (25)
Seronegative spondyloarthropathies, n (%)	12 (20)
Ankylosing spondylitis, n	6
HLA-B27 + (without SA), n	3
Inflammatory bowel disease, n	1
Psoriatic arthritis, n	1
Reactive arthritis, n	1
White dot syndromes,n (%)	14 (23.3)
Birdshot, n	13
Serpiginous, n	1
Other etiologies, n (%)	19 (31.6)
Sarcoidosis, n	6
Vogt-Koyanagi-Harada, n	5
Juvenile idiopathic arthritis	2
Multiple sclerosis, n	2
Pars planitis, n	2
Sympathetic ophthalmia, n	1
IRVAN, n	1
Treatment/s for UME^1^	
Topical, n (%)	2 (3.3)
Local (only), n (%)	14(23.3)
TCA (peri/intraocular), n	5
Dexamethasone implant, n	11
Systemic ± local, n (%)	40 (66.7)
Oral Corticosteroids, n	37
Classic Immunomodulators, n	19
Biologics, n	13
Previous vitrectomy, n (%)	2 (3.3)

^1^One eye may belong to more than one category (four eyes did not receive any treatment). ^2^It refers to qualitatively thickened vitreo-macular interface.

UME, uveitis macular edema; SUN, standardization of uveitis nomenclature classification; SA, spondyloarthropaties; IRVAN, idiopathic retinal vasculitis, aneurisms and neuroretinitis syndrome; TCA, triamcinolone acetonide injections.

**Figure 1:**
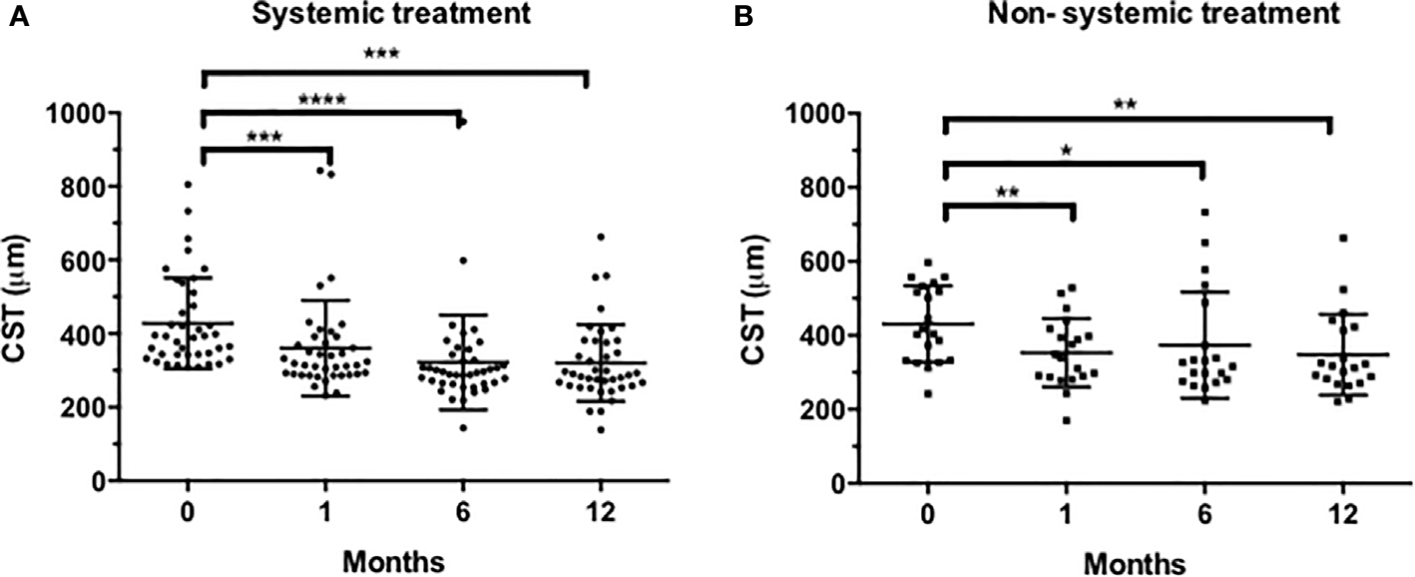
Evolution of central subfield thickness (CST) in systemically treated eyes **(A)** and non-systemically treated eyes **(B)**. Statistical analysis was conducted using the Wilcoxon test (*P < 0.05, **P < 0.001, ***P < 0.001 ****P < 0.0001, vs. baseline).

The overall mean CST was 437.8 ± 122.2 (SD) μm at baseline and decreased significantly after 1 month to 357.4 ± 118.9 μm (P < 0.001), reaching 337.73 ± 135.5 μm at 6 months and 329.6 ± 108.1 μm at 12 months (P < 0.001). Reduction in CST followed a similar pattern in systemically treated patients and non-systemically treated eyes, reaching a significant reduction already at month 1 and maintaining a sustained reduction throughout the 12-month follow-up (**Figure 1**).

Cytokines and Tregs levels were compared between patients with and without systemic treatment. In our cohort, 66.7% of patients had received systemic treatment for their underlying disease (non-infectious uveitis), and 33.3% had not received systemic treatment for at least 6 months before inclusion. There were no significant differences in the levels of cytokines and Tregs between both groups (**Table 2**). However, differences among specific treatments could not properly be evaluated due to the reduced sample size in each specific treatment subgroup.

**Table 2:** Inflammatory mediators in patients with or without systemic treatment at baseline.

Mediator, mean ± SD	No systemic treatment	Systemic treatment	P-value
n (eyes/patients)	20/20	40/40	
TNF-α (pg/mL)	8.83 ± 6.17	9.39 ± 10.44	0.551
IL-1β (pg/mL)	0.91 ± 1.69	0.46 ± 0.52	0.432
IL-6 (pg/mL)	3.10 ± 5.86	6.44 ± 16.43	0.545
IL-8 (pg/mL)	13.66 ± 21.02	12.70 ± 11.57	0.960
IL-10 (pg/mL)	2.01 ± 6.52	0.52 ± 0.97	0.276
IL-17 (pg/mL)	10.7 ± 19.91	15.48 ± 30.68	0.778
MCP-1 (pg/mL)	617.41 ± 404.47	676.45 ± 409.43	0.846
VEGF (pg/mL)	275.58 ± 216.92	226.82 ± 155.29	0.389
Tregs (% CD3^+^ CD4^+^ PoxP3^+^)	3.78 ± 2.46	4.53 ± 2.76	0.392

TNF, tumor necrosis factor; IL, interleukin; MCP, monocyte chemoattractant protein; VEGF, vascular endothelial growth factor; Tregs, regulatory T lymphocytes.

**Figure 2:**
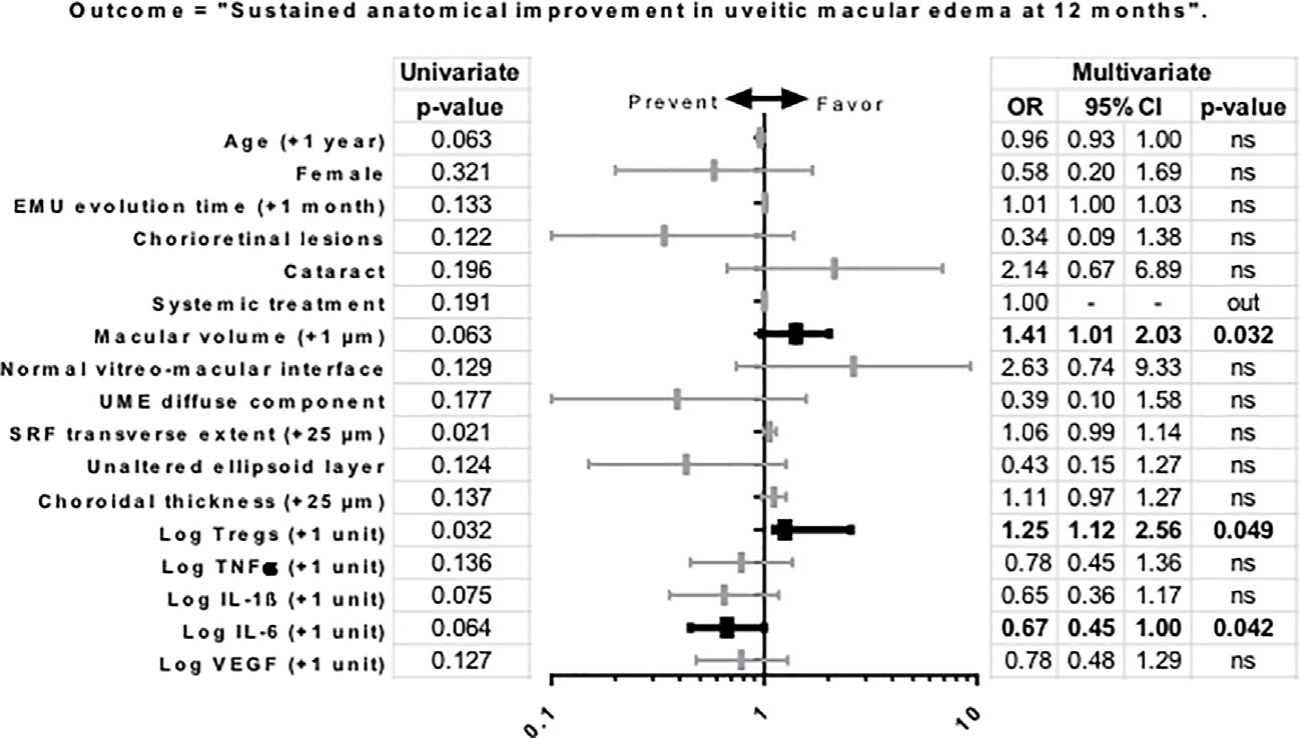
Uni and multivariate regression model of variables as predictors of anatomic outcome in uveitic macular edema. Outcome: “sustained anatomical improvement”, defined as per decrease ≥20% or reaches <300 µm in central subfoveal thickness at 6 months and maintained through month 12. All covariates (P < 0.20) included in the multivariable model are shown. Quantitative variables were transformed to log scale. Statistically significant associations (P-value < 0.05) are noted in bold. Hosmer-Lemeshow goodness-of-fit test, P = 0.13. Abbreviations: CI, confidence interval; OR, odds ratio; UME, uveitic macular edema; SRF, subretinal fluid; TNF, tumor necrosis factor; IL, interleukin; VEGF, vascular endothelial growth factor; Tregs, regulatory T lymphocytes.

In the univariate analysis, qualitatively assessed significant tractional component of the UME was associated to non-sustained anatomical improvement, whereas presence of subretinal fluid was associated to sustained improvement in UME (**Table 3**). To evaluate prognostic factors for UME outcome, variables were analyzed according to the pre-defined CST improvement event with a Step Backwise Logistic Regression Model. Higher macular volume at baseline was the only clinical variable that precluded favorable UME outcome (OR: 1.41, 95% CI: 0.97–2.03, P = 0.032). Interestingly, higher baseline levels of IL-6 were associated with no sustained anatomical improvement of UME at 12 months of follow-up (OR: 0.67, 95% CI: 0.45–1.00); P = 0.042). In contrast, higher baseline levels of Tregs were associated with UME sustained anatomical improvement (OR: 1.25, 95% CI: 1.12–2.56, P = 0.049). The final model showed good goodness-of-fit (Lemeshow P = 0.13) (**Figure 2**). Given the observed associations, the inverse relationship between Tregs and IL-6 was evaluated. Spearman correlation showed no linear association between baseline levels of Tregs and IL-6 (r = 0.254, P = 0.109).

**Table 3:** Clinical variables in UME with and without anatomical improvement.

Clinical variables at baseline		Total	No improvement	Improvement^1^	P-value^2^
Total (eyes/patients)		60/60	38/38	22/22	
Clinical imputs					
Age (years, mean ± SD)		51.1 ± 15.1	54 ± 14.7	46.1 ± 14.8	0.063
Female, n (%)		35 (58.3)	24 (63.2)	11 (50)	0.319
Evolution time (months, mean ± SD)		17.3 ± 31.5	12.5 ± 22.49	25.7 ± 42.2	0.357
Bilateral, n (%)		23 (38.3)	14 (36.8)	9 (40.9)	0.755
ACC (SUN) > 0.5+, n (%)		29 (48.3)	17 (44.7)	12 (54.5)	0.464
Keratic precipitates, n (%)	Fines	10 (16.9)	7 (18.9)	3 (13.6)	0.725
	Granulomatous	6 (10.2)	3 (8.1)	3 (13.6)	
Vitreous haze (NEI) > 0.5+, n (%)		29 (48.3)	20 (52.6)	9 (40.9)	0.381
Chorioretinal lesions (any), (%)		15 (25)	12 (31.6)	3 (13.6)	0.122
Anterior uveitis (SUN), n (%)		16 (26.7)	9 (23.7)	7 (31.8)	0.492
Unclassifiable uveitis, n (%)		7 (20.6)	5 (23.8)	2 (15.4)	0.654
Ocular comorbidities, n (%)	Cataract	16 (26.7)	8 (21.1)	8 (36.4)	0.196
	Glaucoma	11 (18.3)	7 (18.4)	4 (18.2)	0.982
Treatments for UME, n (%)	None	4 (6.9)	4 (10.8)	0 (0)	
	Local only	14 (23.3)	10 (27)	4 (19)	0.191
	Systemic	40 (66.7)	23 (62.2)	17 (81)	
SD-OCT imputs					
Macular volume (mm3, mean ± SD) Thickened vitreo-macular interface, n (%)		11.9 ± 1.9 18(30)	11.5 ± 1.3 14 (36.8)	12.6 ± 2.6 4 (18.2)	0.063 0.129
Unaltered ellipsoid layer, n (%)		35 (58.3)	25 (65.8)	10 (45.5)	0.124
Significant Tractional component, n (%)		7 (11.7)	7 (18.4)	0 (0)	0.032
UME cystoid component, n (%)		47 (78.3)	29 (76.3)	18 (81.8)	0.618
Diameter of major cyst (µm, mean ± SD)		330.1 ± 475.5	266.8 ± 208.8	433.5 ± 723.2	0.706
UME diffuse component, n (%)		14 (23.3)	11 (28.9)	3 (13.6)	0.177
SRF transverse extent (µm, mean ± SD)		363.1 ± 520.2	169 ± 271.6	621.9 ± 665.8	0.021
Choroidal thickness (µm, mean ± SD)		272.8 ± 97.7	258.5 ± 80.6	297.4 ± 119.9	0.137
Immune imputs					
Tregs (% CD3^+^ CD4^+^ PoxP3^+^)		4.2 ± 2.6	4.0 ± 2.3	4.9 ± 2.9	0.153
TNFα (pg/mL)		8.9 ± 8.2	10.1 ± 9.3	7.1 ± 5.9	0.136
IL-1β (pg/mL)		0.7 ± 1.4	1.0 ± 1.7	0.3 ± 0.3	0.075
IL-6 (pg/mL)		4.7 ± 11.9	6.2 ± 14.2	2.3 ± 6.7	0.064
IL-8 (pg/mL)		12.9 ± 18.5	14.7 ± 21.8	10.0 ± 11.1	0.281
IL-10 (pg/mL)		1.5 ± 5.4	1.1 ± 2.4	2.1 ± 8.3	0.281
IL-17 (pg/mL)		12.7 ± 25.4	9.5 ± 14.3	17.9 ± 36.9	0.589
MCP-1 (pg/mL)		645.6 ± 396.3	656.3 ± 410.8	628.4 ± 381.0	0.822
VEGF (pg/mL)		263.8 ± 195.8	282.5 ± 178.8	233.6 ± 221.7	0.127

^1^Improvement, defined as per decrease ≥20% or reach <300 µm in central subfield thickness at 6 months and maintained through month 12.

^2^Chi-square or Fisher (categorical), Mann-Whitney (continuous).

ACC, anterior chamber cells; SUN standardization of uveitis nomenclature; NEI, National Eye Institute scale; UME, uveitic macular edema; SD-OCT, spectral-domain optical coherence tomography; SRF, subretinal fluid; TNF, tumor necrosis factor; IL, interleukin; MCP, monocyte chemoattractant protein; VEGF, vascular endothelial growth factor; Tregs, regulatory T cells.

## Discussion

In this prospective study, we evaluated the association of several inflammatory cytokines and Tregs with anatomical improvement of UME. We analyzed the systemic levels of Tregs and eight serum immune mediators in a large group of patients who presented with macular edema secondary to non-infectious uveitis. Our findings show that high baseline serum levels of IL-6 were associated with poorer anatomical response in UME, whereas high levels of circulating Tregs were associated with UME sustained resolution throughout 12-month follow up.

The involvement of Tregs in controlling inflammation has gained considerable attention in uveitis as well as in other systemic autoimmune conditions (15, 16). To our knowledge, this is the first study that describes the association of Tregs with anatomical outcome in UME. Previous studies have compared Tregs levels between active uveitis, inactive uveitis and healthy control subjects. Some studies reported decreased levels of Tregs in active uveitis compared to inactive or quiescent status (9, 17) or healthy subjects (18), while others observed no differences (19) or even increased levels. Indeed, a previous study by our group showed no differences in Tregs levels between patients with active non-infectious uveitis and healthy subjects (20). Regarding the potential association of UME and Tregs levels, Yeh et al. did not observe differences between uveitis patients with and without UME (9). These discrepancies between studies could partly be attributed to the heterogeneity of uveitis subsets, clinical immunophenotyping and to the different strategies used to characterize Tregs. FoxP3 is the canonical transcription factor for naturally-occurring Tregs and is enriched in human CD4^+^CD25^hi^ T cells. Nevertheless, the CD4^+^CD25^hi^ population does not necessarily capture all FoxP3^+^ cells, therefore we defined CD3^+^CD4^+^FoxP3^+^ as Tregs. The existence of several subtypes of Tregs involved in the modulation of the inflammatory response, could also explain such discrepancies. Thymus originated tTregs inhibit T effector cell trafficking, whereas inducible iTregs primarily prevent T cell priming by acting on antigen-presenting dendritic cells (21). The expression of the transcription factor Helios is a useful marker for the identification of stable tTregs. However, we did not include Helios in our flow cytometry panel to characterize Tregs subtype. Previous studies determined Tregs levels in patients with and without UME in a cross-sectional manner, whereas the present study is prospective in nature, with the aim to investigate the predictive value of Tregs levels in the anatomical response after 12 months.

The differences observed in Tregs levels according to the anatomical event of UME improvement suggest that Tregs could be associated with the protective processes underlying UME resolution. In line with our findings, preclinical evidence shows that in experimental autoimmune uveoretinitis (EAU), the adoptive transfer of Tregs appears to confer protection from uveitis induced by the uveitogenic retinal antigen interphotoreceptor binding protein (IRBP) (22), and another study showed that retinal antigen-specific Foxp3+ Tregs contribute to the natural resolution of EAU and the maintenance of remission (23). Interestingly, Gilbert et al. recently showed that clinical remission of non-infectious uveitis is characterized by an upregulation of peripheral Tregs polarized toward TIGIT and T-bet (24). TIGIT is a co-inhibitory molecule expressed by Tregs. TIGIT^+^ Tregs seem to selectively inhibit Th17 and Th1, but not Th2 responses (25). Since it is known that the Th17 and Th1 subsets are pivotal in the pathogenesis of autoimmune disorders, TIGIT could be a biomarker of stable, functionally suppressive Tregs in such disorders. Even though we did not specifically analyze the suppressive function of Tregs, we observed an independent association between increased Tregs and anatomical improvement or resolution of UME, which was a 25% more frequently found for each unitary increase in Tregs log levels. Almost 67% of recruited patients received systemic treatment showing a good clinical ocular response, supporting the fact that systemic peripheral blood Tregs and their associated microenvironment influence ocular immunity and that, therefore, peripheral blood biomarkers can be useful in predicting ocular outcomes.

On the other hand, the cytokine milieu in non-infectious uveitis has been extensively investigated (26–30). In the present study our focus was investigating the relationship between serum cytokines and anatomical improvement of UME. We therefore selected defined outcomes that may influence UME prognosis and can be easily and objectively assessed. BCVA, although a major clinical end result of UME, is not one of these features, since it can be easily biased by other parameters such as UME duration, presence of cataract, retinal scarring, or macular non-perfusion. We investigated whether anatomical improvement of UME could be predicted by immunological variables besides Tregs after adjusting for covariates in the model. The multivariate model showed that higher baseline levels of IL-6 were associated with lower CST improvement, which was a 33% less frequent for each unitary increase in IL-6 log-levels. IL-6 is a cytokine produced by several immune cells, in response to molecular patterns and affects multiple inflammatory cells and pathways. IL-6 is responsible for the induction of acute-phase proteins, differentiation of adaptive T cell responses, trafficking of acute and chronic inflammatory cells, regulation of homeostasis, and tissue regeneration (31). In the eye, significant elevation of IL-6 has been observed in ocular fluids (vitreous and aqueous) derived from retinal vein occlusion, diabetic macular edema, and refractory/chronic uveitis patients (32–34). Our group and others have previously reported that in patients with UME refractory to conventional therapies, systemic inhibition of IL-6 signaling with the IL-6 receptor (IL-6R) monoclonal antibody tocilizumab (TCZ–Actemra; Hoffmann-La Roche Ltd, Basel, Switzerland) was beneficial at 6, 12, and 24 months (35, 36), in particular with regards to achieving a reduction in CST. Indeed, IL-6 signaling blockade suppresses EAU (37), and it has been recently described that IL-6 reversibly disrupts the integrity of the blood-retinal barrier *in vitro* (38), a key feature of the pathophysiology of macular edema. Our current study actually reinforces the concept of targeting systemic IL-6 in UME, as patients with higher baseline serum levels of IL-6 showed a less favorable anatomical response.

Despite the association of increased Tregs and reduced IL-6 levels with UME anatomical improvement, no linear association between IL-6 and Tregs was observed. The exact relationship between IL-6 and Tregs in modulating uveitis is not clearly understood. IL-6 is involved in the differentiation of CD4^+^ T cells into Th17 cells known to play a pivotal role in uveitis. Moreover, blockade of IL-6 signaling not only suppresses Th17 but also promotes Tregs in EAU (39). In other autoimmune disorders such as psoriasis, IL6 signaling prevents immune suppression by Tregs (40). Other authors have shown that IL6 overproduction *in vivo* inhibits inducible Treg generation from naïve T cells, but does not affect the development and function of natural Tregs (41). The inverse association of IL-6 and Tregs might occur only at the local site within the eye, but not in the systemic circulation, which could explain why we did not observe any association. Alternatively, the limited number of samples could also explain the lack of association. Thus, how IL6 modulates Treg function in the eye warrants further investigation.

Regarding the therapeutic approach, one may expect a relationship between systemic treatment and serum cytokine levels, as immunomodulatory agents are used to treat non-infectious uveitis. However, we did not observe significant differences in cytokine levels between none of the initial treatment modalities, either local, systemic, or a combination of both.

The main limitations of the present study include the sample size of eyes included in the analysis, the heterogeneity of uveitic conditions and immunomodulatory treatments, and the interindividual variability of cytokine levels. Despite these concerns, the strengths of the study include a prospective design, the use of standardized masked data collection protocols, a centralized center for the cytokine and Tregs determination, and the recruitment from multiple uveitis referral centers.

In conclusion, the results presented herein suggest that higher levels of Tregs may contribute to sustained anatomical improvement of UME, whereas increased systemic levels of IL-6 may be a prognostic factor of poor anatomical outcome of UME. These findings could open the opportunity to more efficient and personalized therapeutic approaches to improve long-term visual prognosis in patients with macular edema secondary to non-infectious uveitis.

## Data Availability Statement

The raw data supporting the conclusions of this article will be made available by the authors, without undue reservation.

## Ethics Statement

The studies involving human participants were reviewed and approved by Comité Ético de Investigación Clínica del Hospital Clínic de Barcelona 2013/8574; Comité de Ética de la Investigación con Medicamentos de Euskadi, Hospital Universitario Cruces PI201406; and Comité Ético de Investigación Clínica del Hospital Clínico San Carlos de Madrid 13/244-E. The patients/participants provided their written informed consent to participate in this study.

## Author Contributions

VL, MM, AF, DD-V, AA, and BM contributed to the design of the study and experiments. JM, VL, AF, DD-V, CE, JA, BB, MS, AA, and BM performed the experiments, data capture, and analysis. JM, VL, MF-R, and BM performed the data interpretation. All authors contributed to the article and approved the submitted version.

## Funding

This work was supported by the Ministry of Science and Innovation of Spain, ‘Instituto de Salud Carlos III,’ ‘Fondo de Investigación Sanitaria’ (PI13/00217, PI17/00316, and RD16/0008), and funds FEDER “Una manera de hacer Europa.” We thank the support of the Generalitat of Catalunya (Secretaria d’Universitats i Recerca del Departament d’Economia i Coneixement de la Generalitat, 2017 SGR 0701.

## Conflict of Interest

The authors declare that the research was conducted in the absence of any commercial or financial relationships that could be construed as a potential conflict of interest.
